# Women’s preferences for inpatient and outpatient priming for labour induction: a discrete choice experiment

**DOI:** 10.1186/1472-6963-14-330

**Published:** 2014-07-30

**Authors:** Kirsten Howard, Karen Gerard, Pamela Adelson, Robert Bryce, Chris Wilkinson, Deborah Turnbull

**Affiliations:** 1 Sydney School of Public Health, University of Sydney, Edward Ford Building (A27), Sydney, NSW 2006, Australia; 2 Faculty of Health Sciences, University of Southampton, Southampton, UK; 3 School of Psychology, University of Adelaide, Adelaide, SA 5005, Australia; 4 Obstetrics & Gynecology, Flinders Medical Centre, Adelaide, SA, Australia; 5 Maternal Fetal Medicine, Women’s and Children’s Hospital, Adelaide, SA, Australia

**Keywords:** Induction, Priming, Preferences

## Abstract

**Background:**

In many countries a high proportion of births begin as induced labours. Induction can be lengthy if cervical priming is required prior to induction. This usually occurs as an inpatient, however, an alternative is to allow women to go home after satisfactory fetal monitoring. The aim of this study was to assess the preferences of women for cervical priming for induction of labour in an outpatient or inpatient setting.

**Method:**

A discrete choice experiment (DCE) was conducted alongside a randomised trial of inpatient and outpatient cervical priming (the OPRA trial) in two maternity hospitals in South Australia. 362 participants were included, and women’s preferences for cervical priming for induction of labour were assessed.

**Results:**

Women were willing to accept an extra 1.4 trips to hospital (2.4 trips total) and a total travel time of 73.3 minutes to be able to return to their own home while waiting for the priming to work. For enhanced inpatient services, women were willing to accept a total travel time of 54.7 minutes to have a private room with private bathroom while waiting for the priming to work. The overall benefit score for outpatient priming was 3.63, 3.59 for enhanced inpatient care and 2.89 for basic inpatient care, suggesting slightly greater preferences for outpatient priming. Preferences for outpatient priming increased when women could return to their own home (compared to other offsite accommodation), and decreased with more trips to hospital and longer travel time.

**Conclusions:**

Our results suggest that outpatient priming was slightly more preferred than either enhanced inpatient priming or basic care; these results should be confirmed in different clinical settings. There may be merit in providing women information about both options in the future, as preferences varied according to the characteristics of the services on offer and the sociodemographic background of the woman.

## Background

In western countries such as Australia, the UK and USA, approximately a quarter of births begin as induced labours [1-3]. The induction process can be lengthy if women have an unfavourable cervix as cervical priming is required prior to induction to ripen the cervix. This usually occurs as an inpatient (inpatient priming). Women are encouraged to sleep overnight in the labour ward after satisfactory fetal monitoring, while the priming agent works. Partners are encouraged to go home and the woman is left in an unfamiliar and often noisy and uncomfortable environment.

An alternative option is to allow women to go home (outpatient priming) after satisfactory fetal monitoring and allow them to sleep in their own homes while waiting cervical ripening. The feasibility of outpatient priming or ripening has been explored in several studies [4-9] and is the subject of a recent Cochrane Review [10]. However, there remains a dearth of clinical research evidence, as well as evidence of women’s preferences for alternative models of care, despite both of these aspects being identified as important in recent NICE guidelines [11]. A randomised controlled trial: The Outpatient Priming for Induction of Labour (OPRA) Trial was therefore designed to compare inpatient with outpatient priming [12].

Whilst establishing clinical effectiveness and safety of the options for priming is essential, women’s preferences are also integral to the overall acceptability of the alternatives offered [11]. Those preferences are informed by patient experience factors [13,14] as well as clinical effectiveness. Therefore, information is also needed on what aspects of priming services are important to women, and how these aspects influence the strength of women’s preferences for alternative services. By understanding the relative importance of various aspects of care, and how women make trade-offs between them, we can better design services that are most acceptable to the women who use them.

In the context of cervical priming services, trade-offs between aspects of care delivery such as physical environment and access to medical support may become important. For example, a women receiving outpatient priming goes home and waits in a familiar environment, but if medical attention is needed she accesses it using the phone or by returning to the hospital, whereas a woman receiving inpatient priming is unable to have her partner stay overnight, but has reassurance that if medical attention is needed the doctor and any medical equipment can be readily on hand.

An increasingly used tool for measuring strength of preference for health care is the discrete choice experiment (DCE) [15-18]. Because women often have strong preferences for obstetric care, and the importance of those preferences in decision making, the DCE approach has been successfully used in previous studies of maternity care [19] including intrapatum care [20-22]. However no studies have, as yet, assessed women’s preferences for alternative strategies of cervical priming to induce labour.

The aim of the current DCE is, therefore, to quantify women’s preferences for priming for labour induction and to better understand how women’s preferences can be incorporated into the future development of services.

## Methods

We assessed preferences for alternative models of cervical priming in 362 women being managed at two teaching hospitals in Australia; 260 women were part of a randomised controlled trial (the OPRA trial, see below), and 102 were pregnant volunteers. Specifically we set out to: estimate the strength of women’s preferences for key factors in the patient experience of such services (i.e. physical environment, how care is delivered); establish the trade-offs between factors that may inform how certain configurations of a service might be more acceptable than others and; explore the influence of other factors such as demographic variables on women’s preferences, to better understand the extent to which services may need to be tailored to specific types of patients.

### Participants

The OPRA trial ([23]; http://www.anzctr.org.au) was a 3-year, two-centre randomised controlled trial to compare the effectiveness of : (1) inpatient priming for induction of labour, and (2) outpatient priming with maternal/fetal assessment and CTG monitoring, followed by discharge home, then reassessment the following morning. Trial participants included women scheduled for an induction of labour for social reasons or prolonged pregnancy and who had a healthy term, singleton pregnancy with no medical conditions that would necessitate continuous maternal or fetal monitoring after cervical priming. Cervical priming for both groups was performed with prostaglandin E2 vaginal gels. Trial participants recruited in the second half of the trial received the DCE survey as part of their routine 7-week post-natal questionnaire. In addition, we also sought pregnant women volunteers from the two hospital’s antenatal clinics. Previous evidence has suggested that health care experience can influence preferences, increasing the value that people place on the service [24] and this has been particularly demonstrated in intrapartum care [20,22]. It was therefore important to include a range of experiences with pregnancy and maternity care.

### Discrete choice experiments

Women’s preferences were assessed using a discrete choice experiment (DCE) and followed good research practice [15,16,18]. The DCE approach is based on the idea that goods and services, including health care services can be described in terms of a number of separate attributes or factors and individuals’ value of the service depends on the levels these attributes take. The levels of attributes are varied systematically in a series of questions and respondents answer by choosing the option that they most prefer. The choices selected by the individual result from them weighing up the difference in attribute levels of each choice. From these choices we can estimate how respondents value different attributes across the range of levels presented. Other data collected in the survey, including attitudinal questions and sociodemographic information, can also enter the value functions as explanatory variables.

### Attributes and attribute levels

Based broadly upon the treatment arms in the OPRA trial, alternative priming options were described in terms of six attributes: 1) physical environment while waiting for gels to work, 2) availability of pain relief and sleeping medication 3) the professional who checks while waiting for the gels to work 4) how well the midwife is known to the woman 5) numbers of trips to hospital 6) travel time to nearest hospital offering the service (Table 1). The alternatives are described by this set of attributes, some of which have generic or common levels across outpatient and enhanced inpatient options (travel time and how well the midwife is known) and other which have alternative specific attribute levels (all other attributes) that feature key differences across the alternatives. Specific policy-relevant attributes were developed following qualitative interviews with trial participants who had experienced inpatient or outpatient care [25], the published literature, and an expert panel workshop with obstetricians, midwives and researchers.

**Table 1:** Attributes and levels

**Attribute**	**Levels for outpatient care (Option A)**	**Levels for enhanced inpatient care (Option B)**	**Levels for basic inpatient care (Option C)**
Physical environment while you are waiting for gels to work	**●** Basic accommodation with private bathroom, close to hospital	**●** Twin hospital room with shared bathroom	**●** Twin hospital room with shared bathroom
		**●** Single hospital room with shared bathroom	
	**●** Your own home	**●** Single hospital room with private bathroom	
Availability of pain relief or sleep medication while waiting for gels to work	**●** Single dose of mild pain relief (eg panadeine forte) and sleep medication are provided	**●** Non drug pain relief methods (eg warm baths) are NOT available. Other types of pain relief and sleeping medication available, but may have to wait for doctor for stronger pain medication (eg injection)	**●** Non drug pain relief methods (eg warm baths) are NOT available. Other types of pain relief and sleeping medication available, but may have to wait for doctor for stronger pain medication (eg injection)
	**●** None provided, own pain relief methods can be used, including panadol, warm baths etc.	**●** Non drug pain relief methods (eg warm baths) are NOT available. Mild pain relief (eg panadeine forte) and sleeping medication available from midwives	
		**●** Non drug pain relief methods (eg warm baths) ARE available. Other types of pain relief and sleeping medication available, but may have to wait for doctor for stronger pain medication (eg injection)	
		**●** Non drug pain relief methods (eg warm baths) and mild pain relief (egpanadeine forte) and sleep medication ARE available from midwives	
Who checks on you while you are waiting for gels to work	**●** You can phone a midwife, then come in to the hospital, if you are concerned; if a doctor is needed, they will need to be called in from somewhere else	**●** You will be checked once during the night by the midwife (& woken if you are asleep); a doctor will need to be called in from somewhere else if needed	**●** You will be checked once during the night by the midwife (& woken if you are asleep); a doctor will need to be called in from somewhere else if needed
		**●** You will be checked once during the night by the midwife (& woken if you are asleep); a doctor is available onsite if needed	
	**●** You can phone a midwife, then come in to the hospital, if you are concerned; a doctor is available onsite if needed		
How well you know the midwife	**■** A rostered midwife, with no guarantee that you know her		**●** A rostered midwife, with no guarantee that you know her
	**■** One of a team of 4 or 5 midwives who you have met before		
	**■** One of a pair of midwives who you know already		
	**■** A midwife you already know well		
How many trips you need to make to the hospital	**●** 2 trips	**●** 1 trip	**●** 1 trip
	**●** 3 trips		
The travel time to the closest hospital that provides this service	**■** 10 minutes		**●** 40 minutes
	**■** 20 minutes		
	**■** 30 minutes		
	**■** 40 minutes		

Attribute levels of priming care were presented in three options. These were described as: Option A, an outpatient care option, Option B, an enhanced inpatient care option (including aspects such as private bathrooms and familiar clinicians), and Option C, a fixed basic inpatient care option that described the minimum level of standard care currently available. It was thought not to be clinically appropriate to present an opt-out option. To ensure plausibility of the scenarios, some attribute levels were specific for the alternative presented (Table 1) and we also ensured that the combinations of levels were realistic for the alternatives presented.

### Experimental design

Prior to the main study, a pilot survey was given to 28 pregnant women awaiting clinic appointments and to ten midwives. The pilot survey included 25 questions, with each question offering three options of care (as above). Women reported no difficulties answering the scenarios; some pre-survey instructions were simplified and additional pre-survey explanation was provided for the main study survey. No changes were made to the DCE attributes and levels.

Utilising the parameter estimates from analysis of the pilot study, a statistically efficient [26] fractional factorial design of 50 different choice combinations, segmented into 2 blocks of 25 choices was created using NGENE (http://www.choice-metrics.com) (design d-error of 0.024). Desirable statistical properties were maintained within each block. Some constraints were applied to the design, for example, non-drug pain relief methods such as warm baths were only available when the inpatient accommodation option included a private bathroom. In the final survey, women were presented with 25 choice sets, each with the three alternative options. An example of a choice set is presented in Table 2.

**Table 2:** example DCE question

	**Option A**	**Option B**	**Option C**
Physical environment	Your own home	Single hospital room with private bathroom	Twin hospital room with shared bathroom
Pain relief and sleeping medications, while you wait	None are provided, own pain relief methods can be used including panadol, warm baths, etc.	Non drug pain relief methods (eg warm baths) and mild pain relief (eg panadeine forte) and sleep medication ARE available from midwives	Non-drug pain relief (eg warm baths) methods are NOT available. Other types of pain relief and sleeping medication available but may have to wait for doctor for stronger pain medication eg injection or tablets
Who checks on you while you wait	You can phone a midwife and then come in to the hospital at any time if you are concerned; a doctor is available onsite	You will be checked once during the night by the midwife (& woken if you are asleep); a doctor is available onsite if needed	You will be checked once during the night by the midwife (& woken if you are asleep); a doctor will need to be called in from somewhere else if needed
How well you know the midwife	A midwife you already know well	One of a team of 4 or 5 midwives who you have met before	A rostered midwife, with no guarantee that you know her
Number of trips to hospital	2 trips	1 trip	1 trip
Travel time to the closest hospital that provides these services	10 minutes	40 minutes	40 minutes
*Please pick one*	Choose Option A	Choose Option B	Choose Option C
	□	□	□

The final survey included a description and explanation of the attributes, a practice question to familiarise respondents with the format and interpretation of the attributes, followed by the discrete choice questions and sociodemographic characteristics.

Written informed consent was obtained from all participants in the OPRA trial; for volunteers, consent to participate was implied by completion and return of the questionnaires. The study was approved by the Human Research Ethics Committees of Flinders Medical Centre (Ref: 131/08) and the Women’s and Children’s Hospital (Ref: REC2034/2/11) in Adelaide, Australia.

### Analysis

The data set comprised 25 discrete choice questions of three alternatives for each respondent. A multinomial logit (MNL) model was used to analyse preferences [15,16,18]. The dependent variable indicates the choice from three alternatives in each scenario; this approach is consistent with other discrete choice analyses in antenatal care [19-22]. Utility functions were specified as follows:

$$\begin{array}{ll} V_{\mathrm{Outpatient}\left(\mathrm{OP}\right)}=\beta_{0} & +\beta_{1}\mathrm{PhysicalEnvironment}+\beta_{2}\mathrm{PainRelief}+\beta_{3}\mathrm{StaffCheck}+\beta_{4}\mathrm{KnowMidwife}+\beta_{5}\mathrm{Trips}\\ & +\beta_{6}\mathrm{TravelTime}+\beta_{7}\mathrm{OP}\;\mathrm{Pr}\mathrm{eviousCare}+\beta_{8}\;\mathrm{OP}\;\mathrm{Age}+\beta_{9}\mathrm{OP}\;\mathrm{NESB}+\beta_{10}\mathrm{OP}\;\mathrm{Pr}\mathrm{eviousInduction}\\ & +\beta_{11}\mathrm{OP}\;\mathrm{UniversityEducation}+\beta_{12}\mathrm{OP}\;\mathrm{HealthInsurance}+\beta_{13}\mathrm{OP}\;\mathrm{First}\mathrm{Pr}\mathrm{egnancy} \end{array}$$

$$\begin{array}{ll} V_{\mathrm{Enhanced}\;\mathrm{Inpatient}\left(\mathrm{IP}\right)}=\beta_{14} & +\beta_{15}\mathrm{PhysicalEnvironment}+\beta_{16}\mathrm{PainRelief}+\beta_{17}\mathrm{StaffCheck}+\beta_{18}\mathrm{KnowMidwife}+\beta_{19}\mathrm{Trips}\\ & +\beta_{20}\mathrm{TravelTime}+\beta_{21}\mathrm{IP}\;\mathrm{PreviousCare}+\beta_{22}\mathrm{IP}\;\mathrm{Age}+\beta_{23}\mathrm{IP}\;\mathrm{NESB}+\beta_{24}\mathrm{IP}\;\mathrm{PreviousInduction}\\ & +\beta_{25}\mathrm{IP}\;\mathrm{UniversityEducation}+\beta_{26}\mathrm{IP}\;\mathrm{HealthInsurance}+\beta_{27}\mathrm{IP}\;\mathrm{FirstPregnancy} \end{array}$$

$$V_{\mathrm{BasicInpatient}}=0$$

Models were evaluated for goodness of fit using the likelihood ratio Chi-square statistic for the global test of zero model coefficients, the McFadden’s pseudo R-squared, and Akaike’s information criterion (AIC). The final model was selected on the basis of AIC after testing a number of different model specifications.

Model results are presented as beta parameters, odds ratios and 95% confidence intervals of odds ratios. Trade-offs between attributes were calculated by taking ratios of beta parameters; categorical variables were effects coded. All analyses were conducted using NLOGIT Version 4.0. (Econometric Software, Australia, Castle Hill, NSW, Australia). We also calculated the overall value of alternative priming strategies, using the utility functions and beta parameters from the models, the characteristics of the services as they would be delivered in clinical practice, and representative patient characteristics.

## Results

### Respondents

The majority of pregnant women volunteers (approximately 90%, 102/114) approached agreed to complete the questionnaire while waiting for their clinic appointment. The overall response rate for women in the OPRA trial was 50% (260/515). This yielded a total sample of 362 women (9050 choices), giving an overall response rate of 58%. There were no significant differences in the preference structure of trial participants, compared to clinic women, or across women who were 1) randomised to, or 2) received the different trial arms, so all 362 respondents were included in the analysis [16]. Women were similar to the women in the OPRA trial in terms factors such as of treatment they were randomized to, age, university education and distance from hospital, and the demographic profile was similar to all women who were being induced for prolonged pregnancy at both hospitals (available from authors). Demographic characteristics of the respondents and their experiences of pregnancy and maternity care are shown in Table 3.

**Table 3:** Socio-demographic characteristics and experiences of sample n = 362

	**N**	**%**
Volunteers (hospital clinics)	102	28%
OPRA Trial participants	260	72%
Total participants	362	100%
OPRA Trial Participants (n = 260)^*^		
Randomised to inpatient	132	51%
Randomised to outpatient	127	49%
Age Mean (SD)	29.6 (5.2)	
Non-English speaking background (yes)	41	11%
Highest level of education		
High school	107	30%
Post-high school course	112	31%
Bachelor degree or higher	140	39%
Household combined annual income^2^		
Up to $35,000	53	15%
$35,000 to $65,000	119	33%
$65,000 to $95,000	113	31%
more than $95,000	62	17%
Self-rated health		
Good/very good	325	80%
Neither good nor bad	29	8%
Poor	5	1%
Driving distance to hospital (Median (range))	9 km (1–49 km)	
Estimated travel time (Median (range))	15 minutes (<5 to 45 minutes)	
Private health insurance? (yes)	124	34%
Number of children at time of survey completion		
None (clinic participants only)	55	15%
One	216	60%
Two or more	90	25%
First pregnancy (either during OPRA trial, or currently pregnant with first child (clinic))	239	66%
Type of care with pregnancy/past pregnancies^3^		
Obstetric clinic with doctors	103	29%
Midwives clinics	91	25%
Midwife group practice/birth centre	86	24%
Other	12	3%
Have you ever experienced induction of labour? (yes)	185	51%
Time since giving birth^4^?		
2 months ago	124	34%
3-12 months ago	41	11%
> 1 year ago	88	24%

*randomisation status not available for n = 1 . Missing demographic data for some items (not > n = 3), unless otherwise specified.

% totals not always 100% due to missing data ^2^n = 15 missing data. Australian dollars.

^3^n = 70 (19%) did not answer this question. ^4^excludes currently pregnant and not stated (n = 109, 30%).

### Discrete choice preferences

Preferences were significantly influenced by attributes, and also by some sociodemographic characteristics (see Table 4).

**Table 4:** Women’s preferences for outpatient priming; or enhanced inpatient priming compared to basic inpatient care, (using multinomial logit model)

**Attributes**	**Beta**	**p value**	OR*	**(95% CI)**
**Preferences for outpatient priming (compared to basic inpatient care)**				
Constant	1.051	0.0542		
*Program characteristics*				
Environment while waiting for gels to work	0.572	<0.00001	1.771	(1.445-2.178)
Own home (vs basic accommodation close to hospital)				
Availability of pain relief and sleep medication	−0.029	0.4643	0.972	(0.901-1.050)
Given single dose of mild pain relief and sleep medication (vs providing own pain relief)				
Who checks on you while waiting for gels to work	−0.019	0.7962	0.981	(0.853-1.131)
Availability of doctor on the phone (vs having to wait for doctor to arrive)				
Increasing familiarity with midwife (vs rostered midwife only)	0.095	0.021	1.099	(1.016-1.191)
Extra trips made to hospital	−0.402	<0.00001	0.669	(0.563-0.7960
Travel time for each trip to closest hospital providing service (per minute)	−0.019	0.0005	0.981	(0.971-0.992)
*Patient characteristics*				
Previous care model experienced				
Obstetrics clinic with doctors	−0.815	<0.00001	0.443	(0.331-0.594)
Midwife clinic	0.172	0.165	1.188	(0.935-1.515)
Midwife group practice	0.056	0.6499	1.058	(0.833-1.349)
Age (per year)	0.090	<0.00001	1.094	(1.061-1.128)
Non-english speaking background (vs english speaking)	−1.934	<0.00001	0.145	(0.105-0.201)
Previous experience of induction (vs not)	−0.457	0.0041	0.633	(0.465-0.865)
Highest education -university or college degree (vs not)	0.451	0.0052	1.570	(1.150-2.155)
Private health insurance (vs not)	−0.070	0.5964	0.932	(0.722-1.209)
First pregnancy (vs second or subsequent pregnancy)	0.844	<0.00001	2.325	(1.703-3.190)
**Preferences for enhanced inpatient priming (compared to basic inpatient care)**				
Constant	0.090	0.8519		
*Program characteristics*				
Environment while waiting for gels to work	−0.031	0.4631	0.970	(0.895-1.053)
Private hospital room, but shared bathroom (vs shared room & bath)				
Private hospital room with private bathroom (vs shared room & bath)	0.498	<0.00001	1.646	(1.503-1.805)
Availability of pain relief and sleep medication	0.079	0.0351	1.083	(1.007-1.166)
Stronger pain relief and sleep medication available but may have to wait (vs mild pain relief from midwives), when hot baths not available				
Stronger pain relief and sleep medication available but may have to wait (vs mild pain relief from midwives), when hot baths are available	0.077	0.1746	1.080	(0.968-1.208)
Who checks on you while waiting for gels to work	0.111	0.115	1.117	(0.975-1.282)
Availability of doctor onsite (vs having to wait for doctor to arrive)				
Increasing familiarity with midwife (vs rostered midwife only)	0.130	0.0113	1.139	(1.031-1.259)
Travel time for each trip to closest hospital providing service (per minute)	−0.009	0.0474	0.991	(0.982-1.000)
*Patient characteristics*				
Previous care model experienced				
Obstetrics clinic with doctors	−0.498	0.008	0.608	(0.456-0.813)
Midwife clinic	0.117	0.3419	1.125	(0.886-1.433)
Midwife group practice	0.101	0.4151	1.106	(0.871-1.410)
Age (per year)	0.090	<0.00001	1.095	(1.085-1.105)
Non-english speaking background (vs english speaking)	−0.987	<0.00001	0.373	(0.273-0.511)
Previous experience of induction (vs not)	−0.186	0.2418	0.831	(0.612-1.133)
Highest education -university or college degree (vs not)	0.271	0.0925	1.311	(0.961-1.796)
Private health insurance (vs not)	−0.255	0.0539	0.775	(0.600-1.004)
First pregnancy (vs second or subsequent pregnancy)	0.834	<0.00001	2.303	(1.689-3.156)

McFadden’s R^2^ (pseudo R^2^) = 0.241; Normalised AIC = 1.458.

*Odds of choosing outpatient priming or enhanced inpatient priming (relative to basic inpatient care).

### Preferences for outpatient priming (compared with basic inpatient care)

As would be expected, the ability to return to their own home strongly influenced women’s preferences for outpatient priming. Women were significantly (OR 1.8, 95% CI 1.4 to 2.1, p < 0.0001).

more likely to choose the outpatient care over basic inpatient care when they were able to return home (as opposed to being given off-site accommodation close to the hospital), and when the midwives were more familiar to them (compared to just having rostered midwife care). As the number of extra trips to hospital increased, or as the travel time to the closest hospital providing the service increased, women were less likely to choose the outpatient alternative over basic inpatient care.

The larger negative (beta) coefficient for travel time in the outpatient option indicates that each extra minute of travel time had a larger negative influence on women’s preferences for outpatient care compared with basic inpatient care. The type of pain relief and sleeping medication provided, and the availability of a doctor to talk to on the phone did not significantly influence preferences.

Demographic characteristics of the women, and their past care experiences also influenced women’s preferences for outpatient priming Women whose previous model of care was an obstetric clinic with doctors were significantly less likely to prefer outpatient priming over basic inpatient care, as were women who had previously experienced induction. Older women, women with a university level education and women for whom it was their first pregnancy were significantly more likely to choose outpatient priming over basic inpatient care; while women who came from a non-English speaking background were significantly less likely to prefer outpatient priming over basic inpatient care.

### Preferences for enhanced inpatient priming (compared with basic inpatient care)

The inpatient environment while waiting for priming gels to work influenced women’s preferences. When a private room, with a private bathroom was available, women were significantly (OR 1.6, 95% CI 1.5 to 1.8, p < 0.0001) more likely to choose the enhanced inpatient option over basic inpatient care. Women were also significantly more likely to choose the enhanced inpatient option when the midwives were more familiar to them. When non-drug pain relief methods (hot baths) were not available, the availability of stronger pain relief also became important. As the travel time to the closest hospital providing the service increased, women were less likely to choose the enhanced inpatient option.

Demographic characteristics of the women also influenced women’s preferences for enhanced inpatient priming. Women whose previous model of care was an obstetric clinic with doctors were significantly less likely to prefer enhanced inpatient priming. Older women, and women for whom it was their first pregnancy, were significantly more likely to choose the enhanced inpatient priming option. Women who came from non-English speaking backgrounds were significantly less likely to choose enhanced inpatient priming over basic inpatient care; while education level and previous experience of induction did not significantly influence preferences.

### Trade-offs between benefits and downsides of alternative priming strategies

The trade-offs between potential benefits and downsides of the alternative models of care (compared with basic inpatient care) are shown in Table 5. For outpatient services, women were willing to accept an extra 1.42 trips to hospital (2.42 trips total) and a travel time of 30.6 minutes per trip (73.3 minutes total) to be able to return to their own home while waiting for the priming to work. For enhanced inpatient services, women were willing to accept a travel time of 54.7 minutes (for their one trip to hospital) to have a private room with a private bathroom while waiting for the priming to work. Thus, women were more prepared to be inconvenienced in terms of travel time to have their own home environment, rather than a private room with a private bath (73 minutes vs 55 minutes).

**Table 5:** Tradeoffs between extra trips to hospital and extra minutes of travel time to hospital and other attributes

**Preferred attribute level**	**Number of extra trips women prepared to accept to have preferred attribute level**^**a**^	**Extra travel time per trip women prepared to accept to have preferred attribute level**^**b**^	**Total extra minutes of travel time women prepared to accept to get preferred attribute level**
*Environment while waiting for gels to work*			
Own home	1.42 extra trips (2.42 trips total)	30.6 minutes per trip	73.3 minutes^c^
Private room with private bath	n/a		54.7 minutes^d^
*Familiarity with midwife*			
One of a team of 4–5 midwives met before (vs rostered midwife)			
Outpatient	0.24 extra trips (1.24 trips total)	5.1 minutes per trip	6.3 minutes^c^
Enhanced inpatient	n/a		14.2 minutes^d^
One of a pair of midwives met before (vs rostered midwife)			
Outpatient	0.47 extra trips (1.47 trips total)	10.1 minutes per trip	14.9 minutes^c^
Enhanced inpatient	n/a		28.5 minutes^d^
A midwife that they know well (vs rostered midwife)			
Outpatient	0.71 extra trips (1.71 trips total)	15.2 minutes per trip	26.0 minutes^c^
Enhanced inpatient	n/a		42.7 minutes^d^

^a^βownhome/βtrips; (βmidwifeOP *attribute LevelmidwifeOP)/βtrips.

^b^βownhome/βtraveltimeOP; (βmidwifeOP *attribute LevelmidwifeOP)/βtraveltimeOP.

^c^total trips (column2) *minutes per trip (column3) = total minutes.

^d^βprivateroom&bath/βtraveltimeIP; (βmidwifeIP *attribute LevelmidwifeIP)/βtraveltimeIP.

Similarly, improved familiarity with the midwife was more highly valued (in terms of extra travel time women were willing to accept) in the enhanced inpatient option.

### Overall value of alternative priming options

The overall value based on patient characteristics and beta parameter values (from Table 6) for outpatient priming was 3.63; for enhanced inpatient priming was 3.59, and for basic inpatient care was 2.89; indicating that outpatient primary was slightly more preferred than enhanced inpatient priming for the typical respondent in our survey. Both were preferred over basic care.

**Table 6:** Characteristics of priming services

**Outpatient**	**Enhanced inpatient**	**Basic inpatient (from Table**1**)**
**●** Own home	**●** Private room with private bathroom (8 of 18 labour rooms have a bath)	**●** Twin room with shared bathroom
**●** Single dose mild pain relief and sleeping medication provided	**●** mild pain relief provided, stronger pain relief and sleeping medication available, but may have to wait for doctor	**●** mild pain relief provided, stronger pain relief and sleeping medication available, but may have to wait for doctor
	○ 45% of rooms have a bath (non-drug pain relief available)	○ no rooms have a bath (non-drug pain relief not available)
	○ 55% of rooms do not have a bath (non-drug pain relief not available)	
**●** Doctor available onsite if telephone	**●** Doctor available on site if needed	**●** Doctor will have to be called in from elsewhere if needed
**●** Rostered midwife	**●** Rostered midwife	**●** Rostered midwife
**●** On average 2 trips to hospital (1 extra trip to hospital)		
**Respondent characteristics used (from Table **3**)**		
**●** Median travel time 15 minutes.		
**●** Previous care: 29% obstetrics clinic with doctors; 25% midwife clinic; 24% midwife group practice.		
**●** Mean age 29.6 yrs.		
**●** 11% Non-english speaking background.		
**●** 51% induction experience.		
**●** 39% university educated.		
**●** 34% private health insurance.		
**●** 66% first pregnancy.		

## Discussion

### Main findings and interpretation

The purpose of this study was to determine the preferences of women for inpatient as compared with outpatient priming for induction of labour. Our study found that women prefer outpatient priming and are willing to accept a total travel time of 73 minutes to be able to return to their own home. Other DCE findings have also indicated stronger preference for home-like factors in the delivery of obstetric care [27]. Qualitative studies suggest that women favour outpatient priming [25], that midwives generally have positive views towards outpatient priming [28] and that it is associated with slightly higher levels of satisfaction with care [1,12,29].

Our study also provides additional information on the particular aspects of care, and the trade-offs between these aspects that are likely to be acceptable to women; we have also gained an understanding of the patient characteristics that are likely to drive women’s preferences for alternative priming strategies.

The DCE provides information on the likelihood (as an odds ratio) of choosing a particular option, given changes in attribute levels. However, women are prepared to make trade-offs between levels of attributes, and their own personal circumstances will also influence their decision of what they “really want”. The calculation of a value score for each management option uses the DCE results to try and answer this question, by combining information on the value of individual attribute levels as well as patient characteristics into a single value. The higher overall value score for outpatient priming, suggests that it was slightly favoured over enhanced inpatient care for this group of women. However the differences in scores were not large, and will also be dependent on the characteristics of the women themselves.

As might be expected, preferences for outpatient priming increased when women could return to their own home, and decreased with more trips to hospital and longer travel time. Women’s preferences for inpatient priming increased when a private room with a private bath was available, and decreased with longer travel time.

Sociodemographic characteristics of the women also influenced preferences: older women, and those with a university level education preferred outpatient priming to basic inpatient care. However women whose model of care had been an obstetric clinic run by doctors, and women from a non-English speaking background were significantly less likely to choose both outpatient priming and inpatient priming over basic inpatient care. It is not entirely clear why basic inpatient care would be preferred by these women. It does not appear to be driven by an overall preference for inpatient management, because the enhanced inpatient option was also less preferred than basic care. It may represent some form of status quo preference [30], even though the options were unlabelled; either women had experienced something like this type of care before and preferred what they knew, or perceived it to be usual care, and preferred what they believed was the ‘usual way’ of doing things. Women being cared for by doctors may have a stronger belief in a medicalised model of pregnancy care with inpatient care for direct medical supervision, especially those from non-English speaking backgrounds. It is also possible that some women may have been making assumptions about other factors that were not presented in the scenarios, for example some women may have assumed that the basic inpatient care option was free of charge, and the alternative options were associated with higher costs, even though costs were not included as an attribute. Although our pilot testing indicated that attributes and scenarios were understandable, women who came from a non-English speaking background may have interpreted some of the attributes in unexpected ways. Additional work is needed to better understand these issues, and to ensure that the information provided to women is adequate for them to make informed choices about alternative priming services.

As we did not, contrary to expectations, find differences between women’s preferences as respondents who volunteered compared to those who participated in the trial, it meant the data could be pooled and focus placed on other sources of preference variation. However, it is worth reflecting on possible reasons for why no difference was found. One explanation is that given the nature of the intervention, cervical priming was not required in approximately half the women in each trial arm who gave birth spontaneously. Thus even though women were enrolled in the trial, they did not actually experience induction of labour, and thus the scenarios were presented in the DCE were really hypothetical scenarios rather than experienced.

### Strengths and limitations

This is the first DCE to examine women’s preferences for cervical priming strategy for labour induction. We used a robust method to generate a statistically efficient DCE design and conducted the survey with a large number of participants. We obtained a good mix of respondents with different experiences of labour, induction or other priming strategies by recruiting from trial participants and obstetric clinics at the two hospitals. Although trial participants may not have been representative of women who might use induction services (as indicated by a 50% response rate) we were reassured by the 90% response rate from our clinic volunteers, who did not significantly differ from our trial women concluding that any issues around response rates in trial participants may not be as important in this particular study. It is also less likely that these results are directly applicable to rural and remote patients, where multiple trips to and from hospital for outpatient priming may not be feasible or practical.

## Conclusions

Our results suggest that for the typical patient in our study, outpatient priming was slightly preferred over either enhanced inpatient priming or basic care. In our context, while the clinical outcomes [23] and the costs [31] were very similar between inpatient and outpatient priming, preferences varied according to the characteristics of the services on offer and the sociodemographic background of the woman, suggesting that a one size fits all approach to priming may not be appropriate. For future outpatient priming to be a viable option, it can only be offered to women within the framework of clinical efficacy and safety. These results should be confirmed in other studies in different patient populations and clinical settings; additional research is also needed to establish what choices women would actually make if they were routinely offered a choice as part of clinical care.

## Competing interests

The authors declare that they have no competing interests.

## Authors’ contributions

All authors have made significant contributions to the published study. KH, KG, DT, CW were involved in the conception of the study, KH, KG, PA, DT, CW were involved in the design of the research study. KH, PA were involved in data collection. KH carried out the analyses. KH, KG, PA, RB, CW, DT aided in interpretation of results and revision of the manuscript. KH, KG, PA, RB, CW, DT have all read and approved the final manuscript.

## Pre-publication history

The pre-publication history for this paper can be accessed here:

http://www.biomedcentral.com/1472-6963/14/330/prepub
